# Invisibility of nursing work: technology as a means of combat

**DOI:** 10.1590/0034-7167.202477suppl0301

**Published:** 2024-07-15

**Authors:** Quevellin Alves dos Santos Francisco, Maria Regina Martinez

**Affiliations:** I Universidade Federal de Alfenas. Pouso Alegre, Minas Gerais, Brazil

In the common conception, work is commonly associated with a paid activity in a commercial and legal society, reflecting the modern view that limits it to time exchanged for wage. This perspective, influenced by Industrial Revolution principles, establishes a dichotomy between work and time outside of work, segmenting life into professional and private spheres. However, this approach fails to restrict work’s economic valuation solely to its visibility and ability to generate tangible results, ignoring the non-material production that also emerges from the time invested and is not culturally recognized as valuable^(1)^.

Nursing work, like other activities related to care, tends to be made invisible and devalued. The relationship of care with people escapes management criteria and methods, bringing challenges to its measurement and, consequently, its valorization, even for nursing managers themselves who, for the most part, are male and female nurses: “outside of technical work, there is no work!”^(2)^. Furthermore, the work of producing care comes up against the contemporary neoliberal capitalist context that naturalizes a culture of competitive individualism, compromising the social appreciation of those who provide care.

It is commonplace in our society, i.e., it is not extraordinary or unusual, that those who play essential caring roles are not adequately valued. The act of caring, often dedicated to the most vulnerable, such as children, older adults and sick individuals, challenges the logic of wealth accumulation, requiring investments that, from a neoliberal perspective, are often minimized. The persistence of harmful forms of work organization, which can be exemplified in the context of nursing by the slowness in approving and implementing a wage floor that recognizes the dedicated contribution of these professionals, materializes the challenge of giving more visibility and social appreciation to the profession^(3)^.

Due to the digitalization and virtualization of health systems, activities previously carried out manually are now recorded in a database and analyzed for decision-making^(4)^. This change in the handling of information brings major challenges to the profession, and there is an increase in market expectations for nursing professionals to adapt to the disruption in the traditional way of caring.

Without a doubt, the increasing incorporation of technologies in institutions and services is due to the need to achieve total quality in healthcare. Furthermore, technological innovations have optimized the care provided to patients, as, when they occupy daily healthcare environments, they require a quick response from professionals.

Technological resources’ functionalities guarantee traceability of care and reduced operational costs. Therefore, with its use, nursing professionals can dedicate more time caring for patients and be more effective in the care provided, allowing better care management, increasing work visibility and its consequent social and market appreciation.

The use of technological resources in the act of care by nursing professionals plays a crucial role in combating their invisibility and devaluation, as electronic registration software, mobile device applications, among other emerging resources, guarantee the materialization and more efficient and transparent monitoring of the care provided.

It is time to reflect and overcome the invisibility of the care provided by nursing. Promoting research and discussions on how the profession can handle high technology, and using this to their advantage to seek better positions in the market will allow some current experiences of dedicated and competent professionals to be left in the past, who invest daily in a profession that has offered income incompatible with its social importance and, often, unable to adequately meet their own vital needs.
