# Supplementary material for: Invisibility of nursing work: technology as a means of combat
Source: Rev Bras Enferm. 2024 Jul 15;77(Suppl 3):e77suppl0301. doi: 10.1590/0034-7167.202477suppl0301 (PMC11253833; doi:10.1590/0034-7167.202477suppl0301)
Supplement: Translation

# Translation: Invisibilidade do trabalho da enfermagem: tecnologia como via de combate

**DOI:** 10.1590/0034-7167.202477suppl0301pt

**Authors:** Quevellin Alves dos Santos Francisco, Maria Regina Martinez

**Affiliations:** I Universidade Federal de Alfenas. Pouso Alegre, Minas Gerais, Brasil

Na concepção comum, o trabalho é comumente associado a uma atividade remunerada em uma sociedade mercantil e de direito, refletindo a visão moderna que o limita ao tempo trocado por salário. Essa perspectiva, influenciada pelos princípios da Revolução Industrial, estabelece uma dicotomia entre o trabalho e o tempo fora dele, segmentando a vida em esferas profissional e privada. Contudo, essa abordagem falha ao restringir a valorização econômica do trabalho apenas à sua visibilidade e capacidade de gerar resultados tangíveis, ignorando a produção não material que também emerge do tempo investido e não é reconhecida culturalmente como valiosa^(1)^.

O trabalho de enfermagem, como outras atividades relacionadas com o cuidado, tende a ser invisibilizado e desvalorizado. A relação de cuidado com as pessoas escapa aos critérios e métodos de gestão, trazendo desafios para a sua mensuração e, consequentemente, sua valorização, até mesmo para as próprias chefias de enfermagem, que, na sua maioria, são enfermeiros e enfermeiras: “fora do trabalho técnico, não há trabalho!”^(2)^. Além disso, o trabalho de produzir cuidado esbarra no contexto contemporâneo capitalista neoliberal que naturaliza uma cultura de individualismo competitivo, comprometendo a valorização social de quem cuida.

É banal na nossa sociedade, ou seja, não é extraordinário ou incomum, que aqueles que desempenham papéis essenciais de cuidado não sejam devidamente valorizados. O ato de cuidar, muitas vezes dedicado aos mais vulneráveis, como crianças, idosos e doentes, desafia a lógica de acumulação de riqueza, exigindo investimentos que, sob uma perspectiva neoliberal, são muitas vezes minimizados. A persistência de formas prejudiciais de organização do trabalho, que pode ser exemplificada no contexto da enfermagem pela morosidade na aprovação e implantação de um piso salarial que reconheça a dedicada contribuição desses profissionais, materializa o desafio de dar mais visibilidade e valorização social à profissão^(3)^.

Em razão da digitalização e virtualização dos sistemas de saúde, as atividades antes realizadas manualmente passaram a ser registradas em banco de dados e analisadas para tomadas de decisões^(4)^. Essa mudança na manipulação das informações traz grandes desafios para a profissão, e nota-se um aumento na expectativa do mercado pela adaptação dos profissionais da enfermagem à ruptura no modo de cuidar tradicional.

Sem dúvida, a crescente incorporação de tecnologias em instituições e serviços se dá em decorrência da necessidade de alcançar a qualidade total em saúde. Além disso, as inovações tecnológicas otimizaram o cuidado prestado ao paciente, ao compasso que, ao ocuparem o cotidiano dos ambientes de saúde, exigem resposta rápida dos profissionais.

As funcionalidades dos recursos tecnológicos garantem rastreabilidade dos cuidados e redução de custos operacionais. Portanto, com sua utilização, os profissionais de enfermagem podem dedicar mais tempo cuidando dos pacientes e serem mais efetivos na assistência prestada, permitindo um melhor gerenciamento do cuidado, aumentando a visibilidade do seu trabalho e a sua consequente valorização social e de mercado.

A utilização de recursos tecnológicos no ato de cuidar pelo profissional de enfermagem desempenha um papel crucial no combate à sua invisibilidade e desvalorização, pois os *softwares* de registro eletrônicos, os aplicativos de dispositivos móveis, entre outros recursos emergentes, garantem a materialização e um acompanhamento mais eficiente e transparente dos cuidados prestados.

É tempo de refletir e superar a invisibilidade do cuidado prestado pela enfermagem. Fomentar pesquisas e discussões sobre como a profissão pode manipular a alta tecnologia, e utilizar isso ao seu favor para pleitear melhores posições no mercado permitirá que sejam deixadas no passado algumas vivências atuais de profissionais dedicados e competentes que investem diariamente em uma profissão que tem oferecido rendimentos incompatíveis com sua importância social e, muitas vezes, incapazes de suprir dignamente suas próprias necessidades vitais.

# Translation: Invisibilidad del trabajo de enfermería: la tecnología como medio de combate

**DOI:** 10.1590/0034-7167.202477suppl0301es

**Authors:** Quevellin Alves dos Santos Francisco, Maria Regina Martinez

**Affiliations:** I Universidade Federal de Alfenas. Pouso Alegre, Minas Gerais, Brasil

En la concepción común, el trabajo se asocia comúnmente con una actividad remunerada en una sociedad comercial y legal, reflejando la visión moderna que lo limita al tiempo intercambiado por salario. Esta perspectiva, influenciada por los principios de la Revolución Industrial, establece una dicotomía entre trabajo y tiempo fuera del trabajo, segmentando la vida en ámbitos profesionales y privados. Sin embargo, este enfoque no logra restringir la valoración económica del trabajo únicamente a su visibilidad y capacidad de generar resultados tangibles, ignorando la producción inmaterial que también surge del tiempo invertido y no es reconocida culturalmente como valiosa^(1)^.

El trabajo de enfermería, al igual que otras actividades relacionadas con los cuidados, tiende a invisibilizarse y devaluarse. La relación del cuidado con las personas escapa a los criterios y métodos de gestión, trayendo desafíos para su medición y, en consecuencia, su valorización, incluso para los propios gestores de enfermería, que, en su mayoría, son enfermeros y enfermeros: “fuera del trabajo técnico, hay ¡No hay trabajo!”^(2)^. Además, el trabajo de producir cuidados choca con el contexto capitalista neoliberal contemporáneo que naturaliza una cultura de individualismo competitivo, comprometiendo la valoración social de quienes brindan cuidados.

Es un lugar común en nuestra sociedad, es decir, no es extraordinario ni inusual, que quienes desempeñan funciones esenciales de cuidado no sean valorados adecuadamente. El acto de cuidar, a menudo dedicado a los más vulnerables, como los niños, los ancianos y los enfermos, desafía la lógica de la acumulación de riqueza y requiere inversiones que, desde una perspectiva neoliberal, a menudo se minimizan. La persistencia de formas nocivas de organización del trabajo, que puede ejemplificarse en el contexto de la enfermería por la lentitud en aprobar e implementar un piso salarial que reconozca la contribución dedicada de estos profesionales, materializa el desafío de dar más visibilidad y valorización social a la profesión^(3)^.

Debido a la digitalización y virtualización de los sistemas de salud, las actividades que antes se realizaban manualmente ahora se registran en una base de datos y se analizan para la toma de decisiones^(4)^. Este cambio en el manejo de la información trae consigo grandes desafíos para la profesión, y hay un aumento de las expectativas del mercado para que los profesionales de enfermería se adapten a la disrupción en la forma tradicional de cuidar.

Sin duda, la creciente incorporación de tecnologías en instituciones y servicios se debe a la necesidad de alcanzar la calidad total en la atención sanitaria. Además, las innovaciones tecnológicas han optimizado la atención brindada a los pacientes, que, cuando ocupan entornos sanitarios cotidianos, requieren una respuesta rápida por parte de los profesionales.

Las funcionalidades de los recursos tecnológicos garantizan la trazabilidad de la atención y la reducción de costos operativos. Por tanto, con su uso los profesionales de enfermería pueden dedicar más tiempo al cuidado de los pacientes y ser más eficaces en los cuidados prestados, permitiendo una mejor gestión de los cuidados, aumentando la visibilidad de su trabajo y su consecuente valoración social y de mercado.

El uso de recursos tecnológicos en el acto de cuidar por parte de los profesionales de enfermería juega un papel crucial para combatir su invisibilidad y devaluación, pues *software* de registro electrónico, aplicaciones para dispositivos móviles, entre otros recursos emergentes, garantizan la materialización y seguimiento más eficiente y transparente de la atención brindada.

Es momento de reflexionar y superar la invisibilidad de los cuidados brindados por enfermería. Promover investigaciones y debates sobre cómo la profesión puede manipular la alta tecnología, y utilizar esto a su favor para buscar mejores posiciones en el mercado permitirá que algunas experiencias actuales de profesionales dedicados y competentes que invierten diariamente en una profesión que les ha ofrecido ingresos incompatibles con su importancia social y, a menudo, incapaces de satisfacer adecuadamente sus necesidades, quedar en el pasado.
